# Navigating Recurrence in Clear Cell Renal Cell Carcinoma after Adjuvant Immunotherapy

**DOI:** 10.15586/jkc.v13i3.450

**Published:** 2026-07-03

**Authors:** Goutham Sunny, Shrikanth Ravichandran, Abinash Patnaik, Rajan Yadav

**Affiliations:** 1 Department of Medical and Pediatric Oncology, Gujarat Cancer and Research Centre, Ahmedabad, Gujarat, India;; 2 Department of Medical Oncology, HCG Cancer Centre, Hubli, Karnataka, India

**Keywords:** adjuvant immunotherapy, immunotherapy resistance, recurrence after immunotherapy, renal cell carcinoma, treatment sequencing

## Abstract

Follow-up of clear cell renal cell carcinoma following adjuvant pembrolizumab is a relatively new clinical problem that is influenced by immune pressure and heterogeneous resistance biology. Adjuvant anti-PD-1 treatment has revolutioniz ed postoperative treatment by improving disease-free survival and the overall survival rate but a significant proportion of patients develop recurrence with unique timing, and clinical and molecular pattern, which has important implications. This review attempts to summariz e and explain the available data on recurrence phenotypes, including timing, disease burden, and molecular determinants and then elucidates how these factors influence the choice of subsequent treatment strategies. Early recurrence may suggest primary immune resistance, supporting a mechanism switch towards vascular endothelial growth factor (VEGF)-targeted or hypoxia-inducible factor-2α (HIF-2α)-targeted therapy, although this interpretat ion remains a hypothesis rather than an established mechanistic truth. Conversely, late recurrence may be associated with residual immunological sensitiv ity, potentially permitting immune checkpoint inhibitors–tyrosine kinase inhibitors combinations or immunotherapy rechallenge in carefully selected patients. The status of focal therapy in the oligometastatic relapse, emerging treatment trends in the real world, and recommendations provided by the modern global guidelines are addressed. In the setting where there are no prospective trials in the post-adjuvant setting, a biologically informed, mechanism-based approach is required to streamline sequencing strategies in this unique and more frequently observed population of patients.

## Introduction

Renal cell carcinoma (RCC) has reached a new dimension where adjuvant immunotherapy (IO) provides, for the first time, a significant opportunity to change the natural history of high-risk disease. The historic advantage of pembrolizumab in KEYNOTE-564 (1) was to change the postoperative care and introduce immune checkpoint inhibitors (ICI) as the standard of care for patients who were most potentially to develop recurrence. However, this success has brought about a new and quite common clinical dilemma, namely, what to do when cancer recurs following adjuvant ICI therapy.

Recurrence in this environment is not just a continuation of the normal trajectory of RCC —it is a different biology because of experience of immune pressure. There are tumors that recur after a prolonged interval, raising the hypothesis that residual immunological sensitivity may persist. These conflicting trends have direct treatment consequences, compelling oncology professionals to reenact traditional rules of sequencing therapies and mechanism-switching in metastatic RCC.

No prospective trials are specifically dedicated to this post-adjuvant space, and clinicians must therefore navigate this landscape through extrapolation from mechanistic data, mechanistic reasoning, and an ever-growing body of real-world evidence (RWE). Consequently, the timing and pattern of recurrence have become central to clinical decision-making, guiding decisions on vascular endothelial growth factor (VEGF)-targeted therapy, ICI rechallenge, or novel agents, such as HIF-2α inhibitors. This review aims to clarify the complex biology and management of recurrence after adjuvant pembrolizumab, integrating available evidence to support mechanism-based, clinically informed treatment decisions in a setting where traditional paradigms are evolving.

### Methodology

This narrative review was conducted through a comprehensive search of the published literature using PubMed/MEDLINE and EMBASE, supplemented by conference proceedings from the American Society of Clinical Oncology (ASCO), European Society for Medical Oncology (ESMO), ASCO GU (Genitourinary Cancers Symposium), and European Association of Urology (EAU) annual meetings. Search terms included combinations of “renal cell carcinoma,” “adjuvant immunotherapy,” “pembrolizumab,” “recurrence,” “immune checkpoint inhibitor resistance,” “VEGF-TKI,” “belzutifan,” and “treatment sequencing.” Priority was assigned to phase-III randomized controlled trials, prospective phase-II studies, major international guidelines (EAU and National Comprehensive Cancer Network [NCCN]), and key RWE datasets. Relevant expert consensus statements, including those from Society for Immunotherapy of Cancer (SITC) and the European Delphi panel, were also incorporated. Owing to the absence of dedicated prospective trials in the post-adjuvant relapse setting, a substantial proportion of the evidence reviewed is extrapolated from the metastatic RCC trial populations, acknowledged throughout as a key limitation.

## Rationale and evidence for adjuvant treatment in RCC

It is shown that about 20–40% of patients who have undergone curative nephrectomy for localized RCC still develop recurrence and adjuvant therapy is considered as a way of eliminating microscopic residual disease to enhance disease-free survival (DFS) and the overall survival (OS) (2). Initial adjuvant studies, tyrosine kinase inhibitors (TKIs), did not show any significant benefit. The ASSURE (3), PROTECT (4), SORCE (5), EVEREST (6), and ATLAS (7) trials did not result in significant improvement in terms of DFS or OS and were linked with high toxicity. Even though the S-TRAC trial (8) demonstrated a small DFS improvement with sunitinib (6.8 vs. 5.6 years), there was no OS improvement, and the study was criticized due to its low tolerability. In view of the above-mentioned negative trials, adjuvant TKIs are not routinely recommended outside of clinical trials.

**Table 1:** Major studies that explored adjuvant TKIs.

Study	Treatment arm	Outcome	Conclusion	References
S-TRAC	Sunitinib vs. placebo	Improved DFS, no OS benefit	Positive	Motzer et al. (8)
ASSURE	Sunitinib/ sorafenib vs. placebo	No DFS or OS benefit	Negative	Ravaud et al. (3)
ATLAS	Axitinib vs. placebo	Stopped early	Negative	Gross-Goupil et al. (7)
PROTECT	Pazopanib vs. placebo	Marginal DFS benefit (NSS)	Negative	Motzer et al. (4)
SORCE	Sorafenib vs. placebo	No DFS or OS benefit	Negative	Eisen et al. (5)
EVEREST	Everolimus vs. placebo	Marginal DFS benefit (NSS), no OS benefit	Negative	Ryan et al. (6)

**Table 2:** Major studies that explored adjuvant immunotherapy.

Study	Treatment arm	Outcome	Conclusion	Reference
KEYNOTE-564	Pembrolizumab vs. placebo × 1 year	Significant DFS and OS benefits	Positive	Choueiri et al. (1, 9)
IMmotion010	Atezolizumab vs. placebo × 1 year	No DFS or OS benefit	Negative	Pal et al. (10)
Checkmate 914	Nivolumab + ipilimumab vs. placebo—at least 24 weeks	No DFS or OS benefit	Negative	Motzer et al. (11)
PROSPER	Perioperative nivolumab vs. placebo × 40 weeks	No RFS or OS benefit	Negative	Allaf et al. (12)
RAMPART	Durvalumab alone vs. Durvalumab + tremelimumab vs. placebo	DFS benefits in high-risk population. OS awaited		Staff TAP (13)
LITESPARK-002	ONGOING			

The advent of ICIs has transformed the realm of adjuvant therapy. The KEYNOTE-564 (9) trial showed that pembrolizumab was significantly superior to placebo (DFS hazard ratio [HR] 0.68; overall survival [OS] HR 0.62) for high-risk clear cell RCC (ccRCC) (1). This made it a new standard of care. Other ICI trials have failed to do so; atezolizumab in IMmotion010 (10) and Nivolumab plus ipilimumab in CheckMate 914 (11) were negative, the latter with high-grade toxicity. The discordance between these trials and KEYNOTE-564 may reflect differences in patient selection (inclusion of non-clear-cell histology in some trials), mechanistic differences between PD-L1, PD-1, and dual CTLA-4/PD-1 blockade, and variations in patient risk stratification. Recurrence-free survival did not improve with perioperative nivolumab in the PROSPER trial (12). Early findings of RAMPART trial showed a positive trend in DFS among the high-risk population while the results and publication are still awaited (13). LITESPARK-002 trial is ongoing.

Finally, pembrolizumab is currently the sole adjuvant agent, which is associated with proven DFS and OS benefits in RCC. This highlights the overwhelming importance of the selection of drugs, trial design, and risk stratification of patients.

## Choosing the Right Patient for Adjuvant Immunotherapy

### High-risk population and inclusion criteria for KEYNOTE-564

The KEYNOTE-564 was successful because of the focus ed patient selection and the choice of an effective therapy. The trial solely used patients who had either ccRCC or sarcomatoid ccRCC (100% ccRCC), thus excluding histologic variability, as observed in other negative trials, such as IMmotion010 (10) and PROSPER (14). This guaranteed a biologically homogenous cohort that had higher chances of responding to Programmed Cell Death 1 (PD-1) blockade.

More importantly, M1 NED (no evidence of disease) patients—those with completely resected metastatic disease—the subset at highest risk of recurrence were included in KN-564. This subgroup (5.8% of the participants) was not included in other studies but enriched the trial for relapse events, thereby improving statistical power. Importantly, M1 NED patients are biologically distinct from M0 high-risk patients, having had prior overt metastatic disease that was rendered surgically disease-free; their recurrence biology and therapeutic decision-making may therefore differ from M0 high-risk patients, a distinction warranting consideration when extrapolating treatment strategies.

### Risk models for postoperative survival

Precise postoperative risk stratification has continued to play a leading role in the management of RCC because 20–40% of patients develop recurrence even after a curative resection. There are several prognostic models that are available; however, there is no single universal prognostic system. The tools that are commonly used incorporate clinicopathological factors to provide estimates of risk of recurrence and thereby guide surveillance and adjuvant therapy. The University of California, Los Angeles (UCLA) Integrated Staging System (UISS) (15) integrates tumor, node, and metastasis (TNM) stage, Fuhrman grade, and Eastern Co operative Oncology Group (ECOG) performance status across all RCC subtypes. The stage, size, grade, necrosis (SSIGN) score (16) is a predictor of cancer-specific survival in ccRCC.

One of the most impactful models in the current practice is the Leibovich score (17). It was originally developed and validated to predict recurrence-free survival in localized ccRCC, incorporating tumor stage, nodal status, size, grade, and necrosis, thus classifying patients into low, intermediate, and high-risk groups. It was updated in 2018 and expanded its applicability by including clinical symptoms, sarcomatoid differentiation, perinephric fat invasion, and venous involvement (18). Score of >6 on the Leibovich scale are associated with greater recurrence risk and have adjuvant pembrolizumab candidate selection in expert consensus and trial designs, including RAMPART and SORCE. It should be noted, however, that these clinicopathological models were developed and validated in the pre-IO era. Whether they retain full predictive value in patients treated with adjuvant pembrolizumab or whether novel molecular classifiers are needed to characterize immune-related recurrence risk, remains an important unanswered question.

**Table 3:** Inclusion and exclusion criteria for Keynote-564.

Inclusion criteria	Exclusion criteria
Age: ≥18 years	Presence of residual or unresectable disease at baseline.
Histologically confirmed clear-cell RCC, including tumors with a clear-cell component.	Non-clear-cell histology without a clear-cell component.
At increased risk of recurrence after nephrectomy, based on protocol-defined staging (see below).	Prior systemic therapy for RCC (including targeted agents or IO).
Surgery (radical or partial nephrectomy ± metastasectomy) performed within 12 weeks before randomization, with negative surgical margins.	Active autoimmune disease, immunodeficiency, or requirement for systemic immunosuppression.
M1 NED cohort: patients who presented with resectable metastases (solid, isolated, soft-tissue, non-osseous, and non-brain) that were completely resected at the time of nephrectomy or within 1 year after nephrectomy.	Uncontrolled intercurrent illness or infection.
No prior systemic therapy for RCC.	Brain metastases or osseous metastases (since only non-brain, non-osseous lesions could qualify for M1 NED).
ECOG performance status 0–1 (fully active or ambulatory and capable of light work).	
Disease-free status confirmed at baseline by investigator assessment.	

Notes: ECOG: Eastern Co-Operative Oncology Group; RCC: renal cell carcinoma; IO: adjuvant immunotherapy.

**Table 4:** Definition of high-risk population in KEYNOTE-564.

Category	Stage definition based on 8th AJCC	Details
M0 Intermediate to high-risk	T2, grade 4, or sarcomatoid, N0, M0, or T3, any grade, N0, M0	No nodal or distant metastases.
M0 high-risk	T4, any grade, N0, M0, or any T, any grade, N+, M0	Locally advanced or node-positive disease.
M1 NED	Distant metastasis present at diagnosis (M1), but completely resected to no evidence of disease.	Metastasectomy synchronous or ≤1 year after nephrectomy.

Note: AJCC: American Joint Committee on Cancer.

Hence, KN-564 stands apart as the first successful adjuvant trial in RCC demonstrating significant DFS and OS advantage by utilizing precise high-risk patient selection (T2 grade 4/sarcomatoid, T3–T4, node-positive, or M1 NED) and use of a potent PD-1 inhibitor (pembrolizumab).

## Recurrence after adjuvant treatment

Because a significant number of patients have recurrence after surgery, it is important to know the patterns of recurrence and their effect on subsequent treatment. These can be studied based on three parameters: time to recurrence (TTR), volume of recurrence, and molecular characteristics and biomarkers of resistance.

**Table 5:** Risk models to predict prognosis post-nephrectomy.

Risk MODEL	Year	TNM classification	Grade	T size	Necrosis	Sarcomatoid differentiation	ECOG PS	Clinical symptoms	Tumor thrombus	Perinephric or sinus fat invasion	Extension beyond kidney	References
SSIGN	2002	+	+	+	+	X	X	X	X	X	X	Parker et al. (16)
Leibovich	2003	+	+	+	+	X	X	X	X	X	X	Leibovich (17)
UISS	2004	+	+	X	X	X	+	X	X	X	X	Patard et al. (15)
Leibovich	2018	+	+	+	+	+	X	+	+	+	+	Leibovich et al. (18)

### Time to recurrence

Timing of recurrence is an important prognostic and predictive parameter that provides information on tumor biology and the effectiveness of ICI therapy. No universally validated cut-off time-points have been established. A consensus statement from the Society for Immunotherapy of Cancer (SITC) offers relevant definitions in this context. The timelines for defining resistance vary based on the disease setting in which IO was used (neoadjuvant, adjuvant, or metastatic), whether single-agent or combination IO was employed, and tumor characteristics. Critically, these time-point thresholds have not been prospectively validated and should be regarded as clinically useful approximations, rather than biologically definitive boundaries.

According to SITC, primary resistance or early relapse in the adjuvant setting has been defined as recurrence within 12 weeks of the last dose of IO and those that occur after 12 weeks are termed undeterminable (19). An IO refractory relapse was defined as recurrence during or within 6 months of adjuvant therapy, while standard first line treatment options were unanimously recommended for recurrence after 12 months, according to a European Delphi study. Recurrence that occurred between 6 months and 12 months reflected an area of non consensus among the experts (20). Because pembrolizumab pharmacokinetic data show that PD-1 inhibition can persist for as long as 12 months after a single dose, recurrence that occurs after 12 months may reflect a tumor biology with an initial response followed by an eventual immune escape. In such good biology tumors, based on other characteristics (volume of recurrence and molecular biology), various treatment strategies are considered, including re-treatment with IO (21).

### Volume of recurrence

Although precise definitions of volume are lacking, volume of recurrence remains an important parameter while deciding subsequent treatment strategies. Currently, low-volume disease is operationally defined as an oligometastatic disease not involving the brain, bone or liver, with preserved ECOG performance status (20, 21). While uniform consensus definitions are lacking, objective parameters, such as lesion number (typically 1–5), favorable International Metastatic RCC Database Consortium (IMDC) risk category, and absence of visceral crisis, are used in practice and research. These low-volume recurrences may reflect indolent tumor biology and better clinical outcomes with asymptomatic oligometastatic recurrences being put on active surveillance (20). On the contrary, high-volume recurrences present with significant tumor burden causing symptomatic drop in the ECOG status of the patient. They have poorer outcomes and require aggressive treatment strategies (21).

### Resistance molecular characteristics and biomarkers

Perhaps the most difficult parameter to establish in day-to-day clinical practice among the three parameters, molecular characteristics and biomarkers are used as predictive biomarkers to determine response and probability of relapse. Despite many molecular characteristics having been studied, none is incorporated into routine guidelines because of the lack of reproducibility and prospective validation. Evidence strength varies: some findings derive from exploratory or *post hoc* analyses and none is validated specifically in the post-adjuvant relapse setting. Elevated kidney injury molecule-1 (KIM-1) was associated with higher relapse risk in an exploratory analysis of IMmotion010. Alterations in BRCA1-associated protein 1 (BAP1) and polybromo-1 (PBRM1) are associated with worse and better IO outcomes, respectively, in exploratory analyses of metastatic RCC cohorts (22). A high tumor mutational burden (TMB) and high PD-L1 expression, although considered possible predictors of response to IO, are not adopted into clinical practice because of a lack of consistency in results (21).

Sarcomatoid differentiation is associated with enhanced immunogenicity and is consistently linked with improved responses to IO across multiple metastatic RCC trials. While it may serve as a useful clinicopathological feature to support IO-based strategies, it should not be characterized as a validated predictive biomarker in the post-adjuvant setting where dedicated prospective evidence is absent (21).

## Management of recurrent RCC

Management strategies are organized by recurrence pattern and are necessarily extrapolated from subsequent-line mRCC trials, as no dedicated prospective post-adjuvant relapse trials exist. This extrapolation represents a fundamental limitation that must be acknowledged when interpreting all recommendations in this section.

### Local/focal therapies for oligometastatic disease

In case of oligometastatic recurrence, focal therapy/metastasis-directed therapy (MDT) must never be neglected, particularly in cases where local control is possible. MDT can be done using surgical resection (metastasectomy), stereotactic ablative radiotherapy (SABR), or thermal ablation (23, 24).

#### Surgical resection (metastasectomy)

Surgical resection has been traditionally curative and constantly linked with better OS and prolonged freedom from systemic therapy (25). Retrospective series indicate that 5- and 10-year OS rates might reach 60% and 32%, respectively, after the local recurrence is resected (25).

Positive prognostic variables that are used to select patients ideal for metastasectomy are as follows:

- Complete resection possible.
- Metachronous recurrence ( DFI > 2 years).
- Good performance status.

Ideally, a multidisciplinary team should carry out metastasectomy to balance the risks and benefits against systemic therapy alternatives (21).

#### Stereotactic ablative radiotherapy (SABR/SBRT)

Stereotactic ablative radiotherapy is non invasive and delivers high, curative doses of radiation and is increasingly utilized as an alternative or complement to surgery (23–25). SABR can achieve high local control (LC), which can be as high as 90% after 1 year with minimal high-grade toxicity.

- *SABR in lieu of systemic therapy (systemic-therapy naive)*: In a select group of patients (low tumor burden, good IMDC risk, long DFI), upfront SABR of all lesions can be safely used to delay the start of systemic therapy (23–27). In one phase-II trial, 91.3% of patients who received SABR alone had 1-year freedom from systemic therapy.
- *SABR in oligoprogression*: SABR is useful in the management of a few progressive lesions without replacing an otherwise successful systemic therapy regimen (23–28). This will allow the continuing use of the existing effective agent systemically, and one study has shown that the duration of systemic therapy can be extended by more than 6 months in the majority of patients (28).
- *Active surveillance (AS)*: Asymptomatic oligometastatic disease is a reasonable indication for AS, especially in cases where the recurrence is indolent (21, 24, 29, 30). This practice postpones the use of systemic therapy, which preserves the quality of life and further effective treatment options.

### Non-oligometastatic disease (for systemic treatment)

In patients with polymetastatic disease in need of systemic treatment, the treatment option is dictated by TTR paradigm.

#### TKI alone (mechanism switch)

The initial choice of treatment in refractory/early recurrence (TTR < 12 months) is usually TKI monotherapy. This would help to avoid the probable constitutive immune resistance by targeting the VEGF pathway.

- *Cabozantinib*: Cabozantinib shows inhibitory effects against multiple targets such as VEGFR, c-MET, and AXL, which are sufficient in overcoming the multi-faceted resistance mechanisms that arise in the early recurrence environment (31). The objective response rate (ORR) in trials assessing the use of cabozantinib following failure of ICI was between 30% and 41% with a median progression-free survival (mPFS) of about 8.3–10.8 months (21, 32, 33).
- *Other TKIs*: Axitinib monotherapy following the use of ICI also exhibited an ORR of 45% (34). Tivozanib is a highly selective VEGFR inhibitor, which is also active in the post-ICI environment (35).

#### TKI + IO (combination rechallenge)

This approach is mainly applied in late recurrence (TTR > 12 months) when acquired resistance is suspected, which implies that the immunological system might be re-sensitized to ICI.

Pembrolizumab + lenvatinib: This combination showed superior efficacy in the phase 1b/2 KEYNOTE-146 trial in patients who were treated by means of IO. The ORR in this IO-pretreated population was high at 62.5% and the m PFS was 12.2 months (36). This activity in IO-pretreated populations makes pembrolizumab plus lenvatinib a reasonable option for late recurrence; however, this represents extrapolation from a mixed metastatic population and no direct comparative evidence exists in the post-adjuvant relapse setting.

- *Cautionary evidence*: Two phase-III trials have cast doubt on the strategy of a TKI backbone with an ICI-added back after initial ICI failure:
- CONTACT-03: Atezolizumab (anti-PD-L1) and cabozantinib vs. cabozantinib after ICI failure did not demonstrate improvements in the combination arm (PFS 10.6 vs. 10.8 months, HR 1.03) or OS (25.7 vs. not evaluable, HR 0.94) (37).
- TiNivo-2: The combination arm of Nivolumab and Tivozanib was not superior to tivozanib monotherapy after the failure of the first line of treatment with ICI (35).

Considering the conflicting phase-III findings, the IO/TKI rechallenge option must be applied only using potent and confirmed combinations; specifically pembrolizumab plus lenvatinib under the conditions of late recurrence, after evaluation of due risk.

#### IO–IO or IO re-challenge

Rechallenge with ICI therapy, whether single-agent nivolumab or in combination with ipilimumab (Ipi/Nivo), is generally not recommended in early or refractory relapse. This reflects the current guideline-aligned expert opinion based on the biological rationale of primary immune resistance, rather than an absolute mechanistic contraindication. Centralized analysis of three phase-II salvage studies attempting addition of ipilimumab to nivolumab monotherapy revealed disheartening outcomes with an ORR of 12.6% (38).

Ipilimumab/Nivore challenge is considered in highly selected patients with late recurrence, those with sarcomatoid features, and are physically fit (20, 31, 39). Salvage Ipi/Nivore had an ORR of about 20% and had a rather low m PFS that was 4 months in IO-pretreated cohorts (31).

#### Newer molecules such as belzutifan

Hypoxia-inducible factor alpha (HIF-α ) inhibitor belzutifan has a new, non immune, non-VEGFR mechanism of action that is essential in VHL-mutated ccRCC (2, 31).

- **Monotherapy** (LITESPARK-005): Belzutifan showed a higher ORR (21.9% vs. 3.5%), but no differences in mPFS (5.6 months in both treatments) in a phase-III trial comparing belzutifan with mammalian target of rapamycin (mTOR) inhibitor everolimus in patients who had already received an ICI and a TKI beforehand (40). Regulatory approvals differ by region: in the United States, the FDA has approved belzutifan for previously treated advanced RCC, following an ICI and a VEGFR-TKI. In Europe, the European Medicines Agency (EMA) approval covers patients having received at least two prior lines, including an ICI and two VEGFR-TKIs. Clinicians should consult the current regional prescribing information for the most up-to-date indications.
- Combination (LITESPARK-003, phase II): In this single-arm phase-II trial of IO-pretreated patients, the ORR with belzutifan plus cabozantinib was 31%, m PFS 13.8 months, and median OS 26.7 months (41). These phase-II findings require phase-III confirmation and were not evaluated specifically in a post-adjuvant relapse cohort.

Belzutifan has emerged as an effective therapy in early/refractory disease when the patient needs to undergo a full-scale mechanism switch, or when the patient has progressed on TKI-based regimen.

## Guidelines and real-world evidence

### Current guideline recommendations

The mature OS data strongly support adjuvant pembrolizumab efficacy, and its use is strongly recommended by the EAU RCC guidelines panel (30, 42). The 2026 NCCN Kidney Cancer Guidelines similarly provide a Category 1 recommendation for adjuvant pembrolizumab in eligible high-risk patients, with guidance that post-recurrence therapy should follow first-line metastatic ccRCC standards, acknowledging limited setting-specific evidence. However, recommendations for post-recurrence therapy remain cautious, given the absence of prospective adjuvant-failure data (29).

The EAU panel issued a weak recommendation (explicitly graded as weak) against ICI monotherapy, or combination therapy (IO–IO or IO–TKI) for recurrence within 6 months of adjuvant pembrolizumab. This should be understood as guideline-aligned expert opinion rather than a definitive directive, reflecting the hypothesis that early relapse suggests primary immune resistance, and acknowledge the absence of prospective trial data.

The guideline recommendation in cases of recurrence after at least 12 months of adjuvant ICI is to treat them using the standard-of-care first-line metastatic regimens, and the benefit of using ICI rechallenge cannot be disregarded in the case of such late recurrences (20, 29–31, 42).

### Real-world evidence

The availability of RWE, which is based on multicenter trials and massive databases provide handy background information on the existing clinical practice for the management of recurrence following adjuvant ICI (30, 31, 43).

Preference of TKI: VEGF inhibitors (TKI monotherapy) were used before IO + VEGF inhibitor combinations in patients who had recurrence following adjuvant ICI, which supports the clinical trend of using non immune axis in this specific patient population (20, 31, 43).

Specific agents: Cabozantinib (32–37% overall response) and axitinib were the most-often used treatments in the RWE cohorts of patients who failed IO/TKI (LOT 2+) (44). Cabozantinib is considered as the most preferred choice of TKI for subsequent treatment lines if not utilized earlier (45, 46).

Findings: Individual therapies in RWE of sequential therapy after IO/TKI showed similar clinical benefits of OS and PFS, but the PFS of axitinib monotherapy was worse than that of cabozantinib in multivariate analyses (32–34, 44, 45, 47, 48). The median OS in patients who have received more than two lines of therapy was approximately 17–19.5 months, highlighting the need for new agents to make a meaningful difference in this pretreated population (45, 49, 50).

The RWE substantiates the clinical trend toward VEGF pathway inhibition after adjuvant IO failure, consistent with the biological rationale for mechanism switching. However, the inherent limitations of retrospective real-world datasets —including treatment selection bias, heterogeneity of prior therapy, and variable follow-up—should be acknowledged. These data should therefore be considered supportive and hypothesis-generating rather than practice-defining.

## Conclusion

The time of relapse becomes the most significant aspect to determine the next strategy in recurrent ccRCC after adjuvant pembrolizumab, which is a distinctive therapeutic setting. The demonstrated benefit of adjuvant pembrolizumab on the overall survival makes it a novel standard of care for high-risk patients, but the relapse requires an immediate shift to the strategies that are specific to the disease biology.

Recurrence within 12 months may suggest primary or acquired immune resistance, supporting a mechanism switch to VEGF-targeted agents, such as cabozantinib or the HIF-2α inhibitor belzutifan; these inferences remain biologically plausible but lack prospective validation. Recurrence after 12 months may reflect partial preservation of immune responsiveness, permitting consideration of ICI-TKI combinations or rechallenge in carefully selected patients; pembrolizumab + lenvatinib shows promising activity in IO-pretreated populations, but direct comparative evidence in the post-adjuvant relapse setting is absent.

The use of adjuvant pembrolizumab is strongly recommended for carefully selected high-risk populations. Caution is warranted regarding ICI-based combinations for early recurrences, given the evidence for primary immune resistance. Most critically, dedicated prospective trials enrolling patients specifically in the post-adjuvant relapse setting, incorporating biomarker-driven stratification, are urgently needed. Ongoing studies, such as LITESPARK-011, represent important steps forward, and enrollment in clinical trials should be actively encouraged. Until such evidence is available, all recommendations in this setting represent informed extrapolations from metastatic RCC data and expert consensus rather than evidence-based standards.

## Mandatory AI Declaration

During the preparation of this work, the author(s) used Grammarly and Gemini LLM in order to improve readability and language clarity. After using this tool/service, the author(s) reviewed and edited the content as needed and take(s) full responsibility for the content of the publication.

## Author Contributions

GS and SR conceptualized the idea. GS, SR, RY and AP contributed equally in writing the original draft. It was compiled and reviewed by GS and SR. All authors approved the final version.
